# Quantitative Assessment of Intracellular Effectors and Cellular Response in RAGE Activation

**DOI:** 10.26502/aimr.0168

**Published:** 2024-04-26

**Authors:** Vinitha Deepu, Vikrant Rai, Devendra K. Agrawal

**Affiliations:** 1 Department of Translational Research, Western University of Health Sciences, Pomona, California 91763, USA

**Keywords:** Chronic Inflammation, RAGE, ROS, RAGE assessment, molecular techniques

## Abstract

The review delves into the methods for the quantitative assessment of intracellular effectors and cellular response of Receptor for Advanced Glycation End products (RAGE), a vital transmembrane receptor involved in a range of physiological and pathological processes. RAGE bind to Advanced Glycation End products (AGEs) and other ligands, which in turn activate diverse downstream signaling pathways that impact cellular responses such as inflammation, oxidative stress, and immune reactions. The review article discusses the intracellular signaling pathways activated by RAGE followed by differential activation of RAGE signaling across various diseases. This will ultimately guide researchers in developing targeted and effective interventions for diseases associated with RAGE activation. Further, we have discussed how PCR, western blotting, and microscopic examination of various molecules involved in downstream signaling can be leveraged to monitor, diagnose, and explore diseases involving proteins with unique post-translational modifications. This review article underscores the pressing need for advancements in molecular approaches for disease detection and management involving RAGE.

## 1. Introduction

RAGE is a transmembrane receptor in the immunoglobulin superfamily that plays a crucial role in physiological and pathological processes by recognizing and binding to advanced glycation end products (AGEs) and other ligands [1] Structurally, it has three parts: an extracellular domain for ligand recognition, a transmembrane domain anchoring the receptor in the cell membrane, and an intracellular domain for initiating signaling cascades upon ligand binding [2, 3]. RAGE is expressed on the surface of various cell types, including endothelial cells [4], immune cells [5, 6], and neurons [7]. Its primary function is to mediate the effects of AGEs formed during non-enzymatic glycation of proteins and lipids [8, 9]. Upon ligand binding, RAGE activates various downstream signaling pathways and contribute to cellular responses including inflammation [10], oxidative stress [11–13], and immune reactions.

## 2. Overview of major intracellular signaling cascades activated by RAGE and its clinical implications

**Figure 1:**
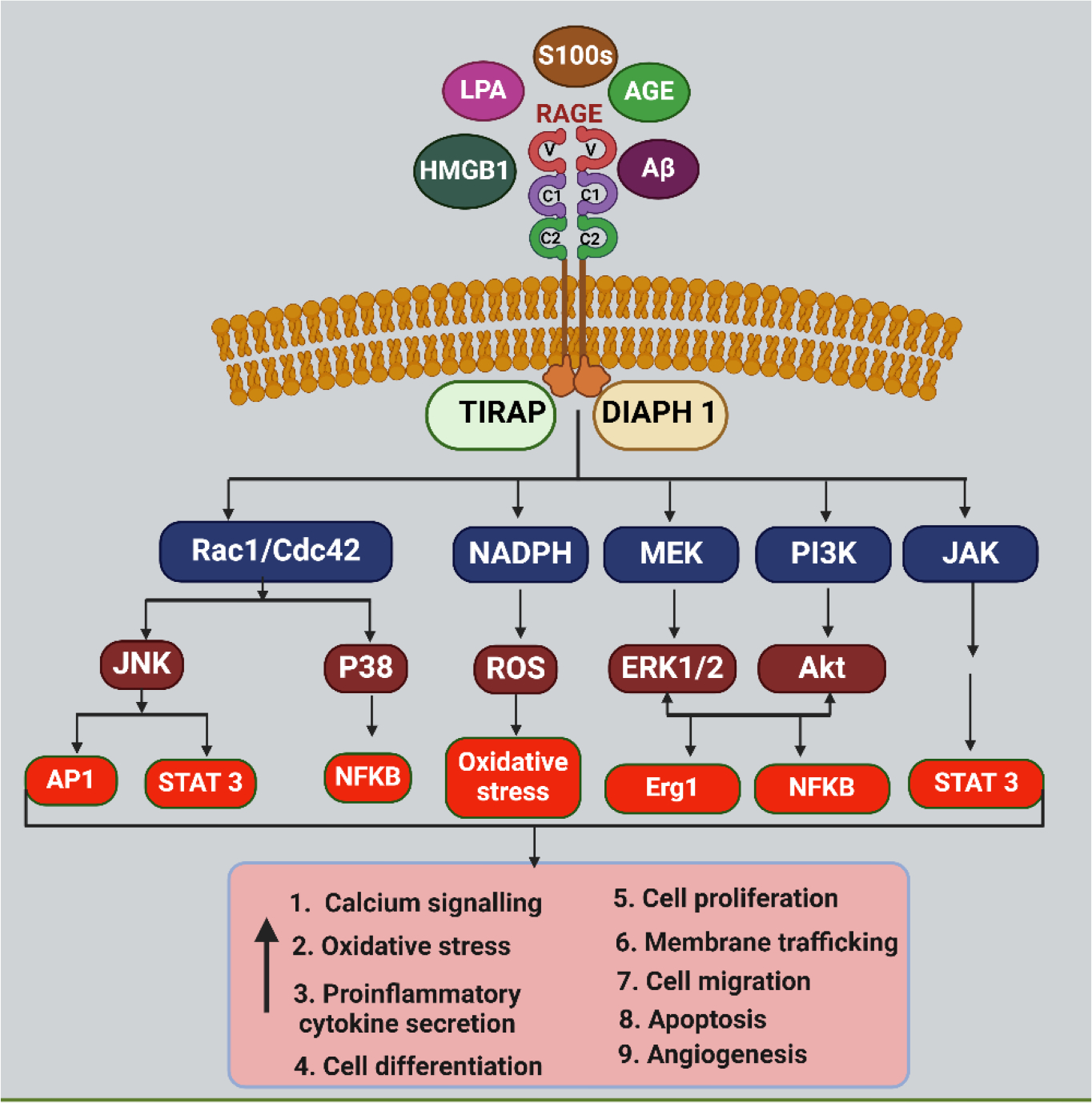
RAGE signaling : RAGE interacts with ligands such as Advanced Glycation End-Products (AGEs), High mobility group box 1 (HMGB1), S100 proteins, Lipoprotein (LPA), Amyloid beta peptide (Aβ), etc to initiate signaling cascades that in turn stimulate Signal transducer and activator of transcription 3 (STAT3), Activator protein 1 (AP-1), Nuclear factor kappa B (NF-κB), and other intracellular transcription factors and activate Nicotinamide adenine dinucleotide phosphate (NADPH) oxidase, Phosphoinositide 3-kinases/protein kinase B (PI3K/AKT), Mitogen-activated protein kinase/Extracellular signal-regulated kinase (MEK/ERK), c-Jun N-terminal kinases/Signal Transducer and Activator of Transcription (JNK/STAT), and Janus Kinase/Signal Transducer and Activator of Transcription (JAK/STAT) pathways. This leads to increased secretion of proinflammatory cytokines which in turn alters cellular activities such as oxidative stress, proliferation, migration, and cell death expression. RAGE primes the cumulative effects of these mediators, leading to chronic inflammation.

**Figure 2:**
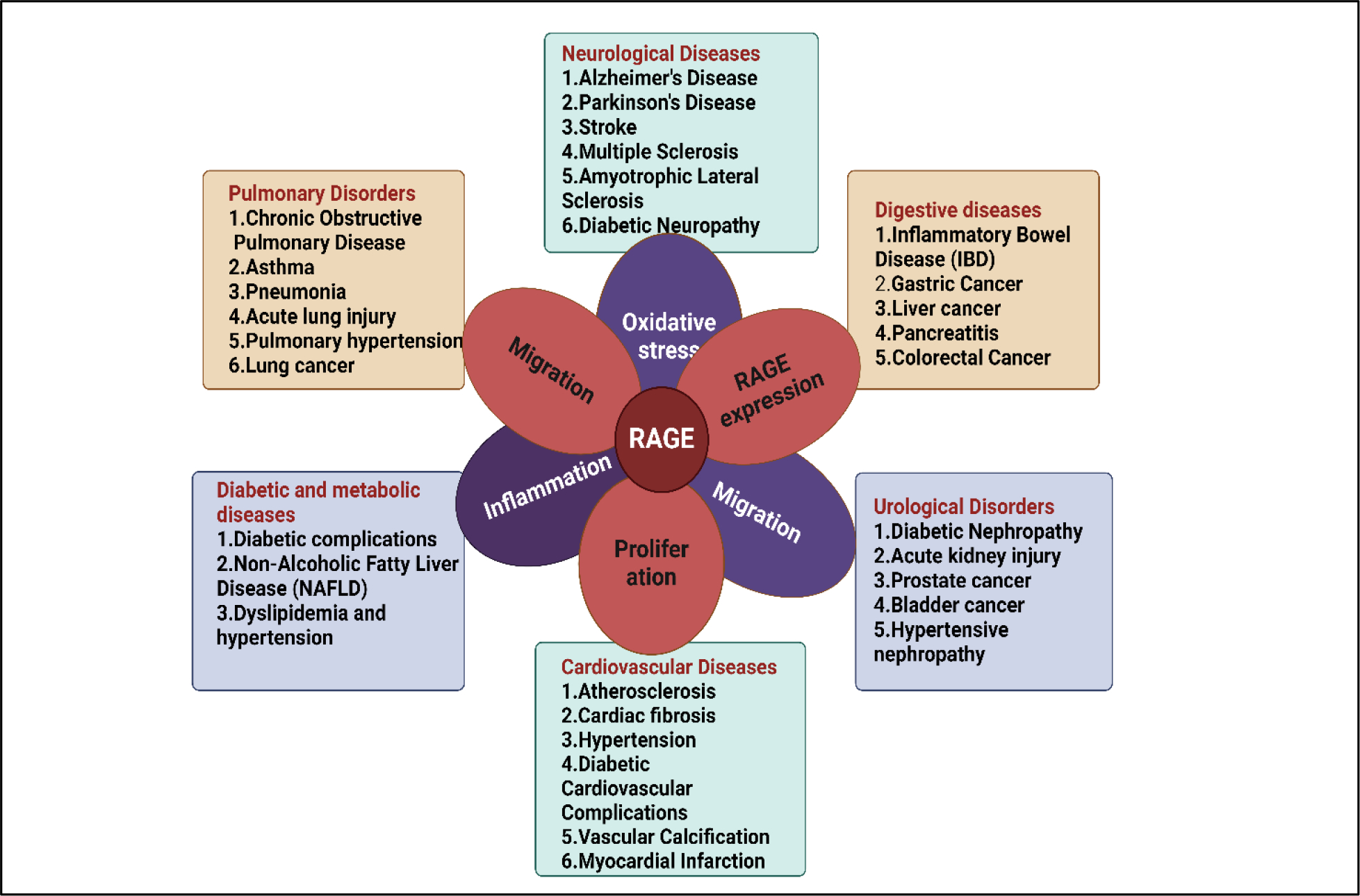
Pathophysiology of RAGE activation in human: Oxidative stress, angiogenesis, proliferation, inflammation, or migration are common mechanisms by which RAGEs are linked to the prevalence of several diseases.

RAGE, a crucial protein in cell signaling pathways, is involved in complex interactions with other cell surface receptors, playing a vital role in cellular responses and protein expression (Figure 1). RAGE activation triggers major intracellular signaling pathways, including the Mitogen-activated protein kinases/Extracellular signal-regulated kinase (MAPK/ERK) pathway [14], Phosphoinositide 3-kinases/protein kinase B (PI3K/Akt) pathway [15], Janus kinase (JAK)-signal transducer and activator of transcription (STAT) [16] pathway, etc. The processes behind RAGE activation are also influenced by its degree of activity. RAGE binding with its ligand phosphorylates its downstream MAPK [17] and activates NF-κB protein and it translocate to the nucleus to stimulate the production of inflammatory mediators through transcription contributing to the amplification of inflammatory signaling. RAGE-mediated activation of PI3K/Akt can influence cell survival and cellular responses [16, 18]. The Janus Kinase/Signal Transducer and Activator of Transcription (JAK/STAT) pathway regulates cell proliferation, differentiation, and immune responses. RAGE-induced activation of JAK/STAT may modulate cellular functions related to inflammation and tissue repair [16]. RAGE activation is associated with the generation of reactive oxygen species (ROS), which contribute to oxidative stress and cellular damage (Figure 2). RAGE-induced modulation of Rho GTPases can influence cytoskeletal dynamics, cell migration, and adhesion, contributing to cellular responses involved in tissue remodeling and inflammation [19].

## 3. Quantitative assessment techniques for measuring RAGE activation in various inflammation

RAGE is widely expressed and linked to a variety of inflammatory-related clinical conditions, including diabetes [50], cancer [15, 51], vascular disease [52, 53], and neurodegeneration [54]. (Figure 2). Many intracellular signaling molecules, including MAP kinases [55], adhesion molecules [56], and transcription factors like NF-κB, Egr-1, AP-1, and STAT3 [57], are activated in response to RAGE activation. The specific downstream effects of RAGE activation can vary depending on the ligand-receptor interaction. For instance, RAGE activation by Aβ is particularly implicated in neuroinflammation and Alzheimer’s disease, while AGEs may have broader implications in various inflammatory conditions [58]. Through their interaction with RAGE, ligands including AGE, HMGB1, and S100s cause endothelial dysfunction, inflammation, oxidative stress, increased vascular permeability, and aberrant angiogenesis, which in turn lead to vascular disturbances. RAGE causes cellular activation in diabetic vasculature due to an increased expression of RAGE and its ligands. Ann Marie Schmidt developed a “two-hit” model for RAGE-mediated alteration of cellular characteristics in diabetes vasculature. According to this model, the diabetic vascular wall exhibits elevated expression of both the receptor (first hit) and RAGE ligands. When a second hit—such as ischemia stress, immunological or inflammatory stimuli, physical stress, or changed lipoproteins—occurs, the body’s reaction is heightened, which leads to the development of vascular lesions rather than the restoration of vascular homeostasis [59]. In a number of clinical situations, such as diabetes [60], chronic inflammation and malignancies [61], and neurodegenerative diseases [62], RAGE is linked to enhanced host responses such as second hit. The clinical implications of RAGE are listed in Table 1

The gene and protein expression of the signaling molecules in downstream signaling and the downstream effectors of RAGE such as NF-κB [63], STAT3 [63], AP1 [64] and Erg 1[10] may be investigated using a variety of conventional techniques including ELISA, RT-PCR, Western blotting, and immunostaining, however, there are limitations and will be discussed in sections below. Western Blotting, particularly, fluorescent multiplex analysis using western blotting allows the detection and quantification of RAGE protein in inflammatory responses by detecting alterations in RAGE protein levels and post-translational modifications (PTMs) [65]. Particularly during inflammatory events, PTMs are essential for controlling the activity and function of a protein [66] RAGE endures post translational modifications such as phosphorylation [67], glycosylation [68], ubiquitination [69], sumoylation [70], acetylation [71], nitrosylation [72], palmitoylation [73], O-GlcNAcylation [74], and proteolytic cleavage [71] during inflammatory events. These changes are crucial targets for therapeutics meant to modulate RAGE activity because they add to the dynamic character of RAGE signaling and its participation in several inflammatory diseases.

Accurately quantifying and comparing expression levels might be difficult because of the isoforms of RAGE such as soluble versions (sRAGE) and membrane-bound full-length RAGE. These isoforms must be considered when studying RAGE expression using various techniques. It might be difficult to find and use species specific antibodies that are specific for identifying RAGE and its isoforms. Thorough antibody validation is necessary to provide accurate findings. Distinct tissues and cell types may have distinct factors regulating the expression of RAGE [75]. Inflammation and oxidative stress are two pathogenic factors that cause dynamic regulation of RAGE expression [76]. This suggests that ligand mediated RAGE activation may activate downstream signaling differently in each pathological condition to have its effects at the molecular and cellular level. This differential expression of these downstream mediators and the extent of RAGE expression should be assessed in different pathogenic conditions using precisely designed investigating techniques. RAGE-ligand interaction activate intracellular signaling including NADPH oxidase, PI3K/AKT, MEK/ERK, SAPK/JNK, and JAK/STAT and transcription factors like NF-κB, Egr-1, AP-1, and STAT3. An increased RAGE expression results in modifications in the expression of proteins and changes in cellular processes, such as inflammation, oxidative stress, angiogenesis, proliferation, and migration.

### 3.1 Quantifications of post translational medications

Immunoblotting allows for the simultaneous detection of multiple protein expressions occurring during PTMs, providing a comprehensive view of post-translational landscape of various proteins [77]. It is versatile and allow it to be applied to various samples and distinguish different isoforms or variants of a protein. Nevertheless, immunoblotting is regarded as semi quantitative, and other methods could be needed for exact quantification. The quality and specificity of the antibodies used determine how specific the results are, and cross-reactivity with other nonspecific proteins may happen. Particularly for proteins with very close molecular weights or its isoforms, immunoblotting may have low specificity, making it challenging to discern minute alterations [78].

For increased specificity, precision, and repeatability, immunoblotting assays for various proteins can be improved by implementing modern multiplexing technologies, automation, quantitative methodologies, optimal sample preparation, and thorough antibody validation [79]. Such methods can be integrated with automated procedures, digital imaging, densitometry analysis, and better resolution techniques [80]. The post-translational modification proteins of RAGE can influence its ability to bind various ligands, including advanced glycation end-products (AGEs) [71], high-mobility group box 1 (HMGB1)[81], and S100 proteins [71].

### 3.2 Differential gene expression analysis of RAGE and its downstream effectors

The molecular processes behind the activation and activity of RAGE and its effector proteins can be understood through delineating changing gene expression during pathogenesis of various pathological conditions. It is particularly crucial when it comes to disorders like diabetes, inflammation, neurological illnesses, and problems with the cardiovascular system. Gene expression studies, particularly those using high-throughput techniques like RNA-seq, provide a comprehensive analysis of the entire transcriptome [82]. Transcriptomic analysis will also help in revealing how gene expression patterns of various molecules in downstream signaling of RAGE change over time in response to RAGE activation. This information is crucial for understanding dynamic biological processes and correlating gene expression data with other omics (e.g., proteomics) for a more comprehensive understanding will be useful in developing novel therapeutics.

#### 3.2.1 Drawbacks of gene expression analysis

In some way, understanding the gene expression profiles of RAGE and its effectors can help identify potential therapeutic targets, aiding in the development of intervention strategies to modulate RAGE-associated pathologies. Gene expression studies also contribute to understanding inter-individual variability [83] in RAGE-related responses, advancing personalized medicine approaches and aiding in the early detection and prediction of disease outcomes. Thus, RAGE-related genes can be used for targeted drug development and optimization. However, there are various challenges in gene expression analysis. RNA-sequencing can been used to identify genes that are differently expressed upon RAGE activation both in vitro and in vivo. Through the analysis of transcription factors and pathways regulated in the presence of RAGE activation, the RNA-seq data offer an insights into potential strategies of suppressing RAGE-mediated inflammation [84].

RNA sequencing does not give single nucleotide polymorphisms (SNPs)[85] or the profile of gene expression from introns; it only provides information from exons [86]. Particularly in samples with low RNA content or complicated gene expression patterns, it might overlook uncommon splice variants or low abundance transcripts, restricting the transcriptome ability to be thoroughly analyzed [87]. Furthermore, precise transcript isoform modeling and quantification from RNA-seq data can be difficult, especially in areas with complicated gene structures [88], alternative splicing processes [89], or transcript overlap [90]. Because RNA-seq only captures a snapshot of gene expression at a particular moment in time, it is not able to record dynamic changes in regulatory mechanisms or gene expression across time or in response to external stimuli [91]. Recent developments in single-cell and single-molecule imaging technologies have allowed us to resolve biological processes in space and time that are essential for comprehending how genes are expressed [92]. Highly dynamic elements of transcriptional and post-transcriptional regulation in eukaryotic cells have been discovered by observations of single-molecule processes in their cellular environment. Using this method, transcription may be linked to the amount and lifespan of mRNA [92].

Other drawbacks could include the requirement for standardized RNA sizes, the standard size selection of RNA-Seq libraries making small transcripts more challenging to count, the possibility of transcript overlap between two different genes, and the potential for transcript-length bias resulting from RNA-Seq multiple fragmentation and cDNA or RNA size-selection steps[82]. ATAC-seq, a sensitive method with the ability to map open chromatin in a small number of cells, when integrates with RNA-seq elucidate more specific gene regulation involved in the pathogenesis. Identifying open chromatin regions via ATAC-seq unveils potential regulatory elements like enhancers. RNA-seq complements by quantifying active gene expression levels. This integration links accessible chromatin with transcribed genes, highlighting regulatory mechanisms. Notably, when active genes align with accessible chromatin, it suggests regulatory element presence, influencing gene expression. Thus, ATAC-seq/RNA-seq synergy unveils the intricate relationship between chromatin accessibility, regulatory elements, and gene expression. However, utilizing RNA sequencing for gene expression studies presents a number of difficulties. The approach is skewed towards polyadenylated transcripts, potentially excluding non-polyadenylated RNAs including microRNAs, long noncoding RNAs (lncRNAs), and circular RNAs, which demand the use of specific library preparation techniques to identify [93]. In order to examine gene expression patterns at the single-cell or subcellular level, conventional RNA-seq approaches lack the spatial resolution necessary to yield bulk transcriptome measurements, which average gene expression over whole tissues or cell populations.

### 3.3 Importance of intracellular calcium dynamics quantifications on RAGE activation

Activation of RAGE can modify calcium signaling via several methods, which can change cellular responses and play a role in the development of several diseases. Extracellular calcium influx is promoted by the binding of RAGE to its ligands, such as S100 proteins, through a variety of calcium-permeable ion channels, including transient receptor potential (TRP) channels [94]. RAGE activation raises intracellular calcium levels, which in turn affects the activity of calcium-dependent signaling pathways, including calcineurin-nuclear factor of activated T cells (NFAT) and calmodulin-dependent protein kinase (CaMK) [95]. When RAGE is activated, these pathways can control gene expression, cell division, apoptosis, and inflammation [96]. Quantitative measurement of intracellular calcium dynamics using fluorescent indicators provides valuable insights into the role of RAGE in calcium signaling and associated cellular responses. By enabling the quantitative assessment of calcium kinetics, they make it possible to compare different cell types or experimental conditions. When examining RAGE activation in various biological conditions, fluorescent indicators are a flexible tool that provide insights into cellular variety by seeing the variation in calcium responses across individual cells.

β-amyloid [72], pathological oxidation protein products [73], and advanced glycation end products all activate the signal receptor RAGE [74] and leads to the activation of various protein kinases like phospholipase C (PLC) [97]. When the PLC is activated, inositol trisphosphate (IP3) is produced [98]. When IP3 binds to IP3 receptors on the endoplasmic reticulum (ER), calcium ions are released into the cytoplasm [99]. On the plasma membrane of neurons and astrocytes, RAGE activation can also result in the activation of calcium-permeable ion channels, such as transient receptor potential (TRP) channels [100]. Gliotransmitters like glutamate are released exocytotically from presynaptic terminals of neurons when intracellular calcium levels rise. Glutamate causes synaptic transmission and neuronal excitability via binding to postsynaptic receptors [100]. This particular pathway plays a role in the communication between neurons and astrocytes in both physiology and disease [101]. Fluorescent markers such as Fluo-4 AM or Fura-2 AM, can be used to quantitatively assess the intracellular calcium dynamics upon RAGE activation by specific RAGE ligand like AGEs or S100 proteins [102]. RAGE can trigger intracellular signaling cascades, such as calcium signaling, by attaching itself to S100 proteins. The interaction between S100 proteins and RAGE can cause calcium to enter the cytoplasm via activating calcium channels or releasing calcium from intracellular reserves, among other ways [103]. S100 proteins are calcium-binding proteins [104]. Elevated calcium levels in the cytoplasm initiate subsequent signaling pathways, which involve the initiation of calcium-dependent enzymes like Calcineurin, Phospholipase C (PLC) and Protein kinase C (PKC), protein kinases such as MAPK, JNK, Akt etc, and transcription factors like NFKB, STAT3, AP1 etc [105]. Numerous biological functions, including as gene expression, cell division, proliferation, migration, and apoptosis, are regulated by these signaling pathways.

Altered calcium levels can provoke significant pathological consequences across multiple bodily systems[106]. Hypocalcemia, characterized by low calcium levels, can trigger muscle tetany [107], neurological disturbances like seizures[108], cardiac arrhythmias[109], and bone demineralization [107]. Reduced calcium availability compromises conduction, excitability, and contractility in cardiac cells, leading to arrhythmias. Prolonged QT intervals increase the risk of dangerous ventricular arrhythmias [110]. These disturbances in electrical activity pose significant risks in cardiovascular diseases, potentially culminating in life-threatening events. Conversely, hypercalcemia, elevated calcium levels, may induce muscle weakness [111], kidney diseases [112], cognitive impairment [113], and cardiovascular complications including arterial calcification [114]. Elevated calcium levels impair endothelial function [115], induce smooth muscle cell differentiation into calcifying cells [116], trigger matrix vesicle formation [117], exacerbate inflammation, and inhibit vascular calcification inhibitors. Increased intracellular calcium levels triggered by inflammatory mediators lead to adherent junction disassembly, cytoskeletal rearrangements, and increased permeability. Additionally, calcium signaling facilitates leukocyte-endothelial interactions, including rolling, adhesion, and transendothelial migration during inflammation [115]. Smooth muscle cell differentiation into calcifying cell is a crucial event in vascular calcification associated with atherosclerosis. Increased intracellular calcium levels, triggered by osteogenic stimuli, promote osteogenic gene expression and matrix calcification [116]. Elevated calcium levels stimulate vesicle release from vascular smooth muscle cells, leading to calcium phosphate deposition and hydroxyapatite crystal formation. Matrix vesicle formation promotes vascular calcification, plays a potential role in the progression of CVD [117]. These mechanisms promote calcium deposition in arterial walls, contributing to atherosclerosis, arterial stiffness, and increased CVD risk. In neurodegenerative diseases like Alzheimer’s, dysregulated calcium signaling contributes to neuronal dysfunction and degeneration. Moreover, calcium imbalances underlie conditions such as osteoporosis, where diminished bone density increases fracture risk. These disruptions also reflect calcium’s pivotal role in muscle contraction, neurotransmitter release, cell signaling, and bone integrity. Understanding the intricate interplay between calcium levels and pathological outcomes underscores the necessity for precise calcium homeostasis maintenance to safeguard overall physiological function and prevent the onset of various diseases. It is possible to record and analyze variations in intracellular calcium levels by using a fluorescence microscope to see the florescence emission of calcium indicator. It is essential to conduct studies using calcium chelators, such as ethylene glycol tetra acetic acid, in order to verify if the observed changes in fluorescence are dependent on calcium [118]. It makes it possible to track intracellular calcium dynamics in real-time. With the ability to resolve changes in calcium inside individual cellular compartments spatially, fluorescent calcium indicators provide a dependable way to measure intracellular calcium levels [119].

Different domains of the extracellular component of RAGE are interacting with RAGE ligands, such as S100 proteins such as S100A8, S100A9, and S100B, to have different biological effects. S100A2 exhibited severe calcium dependence and a micromolar affinity for RAGE in vitro. Additionally, S100A2 interacts with the receptor’s V-domain, and its binding to GST-RAGE is only possible in the presence of calcium [120]. Cardiovascular disorders including hypertension and atherosclerosis are characterized by endothelial dysfunction and vascular inflammation, which are exacerbated by S100A8-RAGE-mediated calcium signaling [121]. Neuroinflammatory processes and neurodegenerative disorders like Alzheimer’s disease are linked to aberrant calcium signaling that is triggered by RAGE-S100A9 interactions [122]. Excitotoxicity, mitochondrial malfunction, and synaptic impairment are some of the effects of calcium influx into neurons that can cause harm to neurons and ultimately result in cell death [122].In several cancer types, RAGE-S100B-induced calcium signaling promotes tumor development and metastasis [123]. Invasion, migration, resistance to apoptosis, and proliferation of cancer cells are all accelerated by calcium entry, which aids in the growth of tumors [123]. Poor prognosis, metastatic dissemination, and aggressive tumor characteristics are linked to RAGE-S100B-induced calcium signaling.

#### 3.3.1 Drawbacks and limitations with quantitative intracellular calcium dynamics measurement

Although it makes it possible to monitor intracellular calcium dynamics in real time, there are a few drawbacks and difficulties in quantitatively measuring intracellular calcium dynamics. The number of fluorescent markers that are loaded into cells varies depending on the cell density and dye loading parameters. High-affinity dyes may alter cellular activity through their cytotoxic effects [119]. Dye may cause artifacts or interfere with biological processes, and real-time information may be restricted [124]. To be confident that fluorescence variations are connected to calcium dynamics, careful confirmation is required. Because calcium ions function as second messengers in a variety of physiological activities and are involved in several signaling pathways, fluorescence changes are linked to calcium dynamics. The amount of calcium ions in a cell could be measured using calcium-sensitive dyes, which offers important insights into the dynamics of calcium [125].

Single-cell resolution has its benefits, but there may also be a drawback when examining tissues or intricate cellular networks where calcium signaling may be influenced by connections between cells [126]. The limitations of quantitative intracellular calcium dynamics measurement are signal saturation, dye leakage, photobleaching, and background fluorescence, as well as spatial heterogeneity within cells. These limitations can be mitigated by using membrane-permeant acetoxymethyl (AM) ester forms of calcium dyes to reduce dye leakage, optimizing dye loading protocols and minimizing dye exposure time, and diluting the calcium-sensitive dye or using lower dye concentrations. Additionally, using ratiometric dyes can provide a more reliable indicator of calcium concentration. To reduce photobleaching, lower excitation intensities, shorter acquisition times, and intermittent illumination protocols can be used [127]. To overcome spatial heterogeneity, high-resolution microscopy techniques can be used to provide spatial information on calcium dynamics within subcellular compartments. Complementary approaches such as ratiometric imaging, high-resolution microscopy, and advanced image analysis techniques can provide valuable insights into intracellular calcium dynamics with improved spatial and temporal resolution [128].

### 3.4 RAGE: Intracellular calcium signaling and increased oxidative stress.

Intracellular calcium levels rise as a result of signaling cascades triggered by ligand (AGE/S100s) binding to RAGE [129]. Elevated intracellular calcium can activate calcium-dependent signaling pathways, including those that control gene expression involved in various pathological conditions. Calcium functions as a secondary messenger in a variety of signaling pathways [130] and calcium signaling pathways have the ability to join on transcription factors that are involved in the transcriptional control of genes, including RAGE, such as CREB (cAMP Response Element-Binding) [131]. ChIP assay (Chromatin Immunoprecipitation assay) identifies target genes that are directly controlled by RAGE in inflammation by establishing a correlation between RAGE binding to particular genomic areas and changes in gene expression levels. Additionally, information on chromatin remodeling activities connected to RAGE-mediated transcriptional activation or repression of inflammatory genes such as such as NF-κB, AP-1, or STAT3, obtained using histone modification ChIP tests (e.g., ChIP for histone H3 acetylation or methylation) [132].

Inflammatory diseases are frequently linked to calcium signaling and ROS generation [133]. The relationship between these mechanisms in context of RAGE expression could increase inflammatory reactions and contribute in the development of inflammatory disorders [134]. In cases of cellular stress, RAGE expression is elevated, and two essential elements of cellular stress responses are the generation of ROS and calcium signaling [135]. Cellular responses that are either maladaptive or adaptive may be influenced by the interactions between these systems. The production of ROS occurs from RAGE activation via a number of routes, such as the stimulation of oxidative stress pathways, mitochondrial malfunction, and NADPH oxidase activation [10]. ROS have the ability to enhance calcium signaling by promoting calcium inflow and release from intracellular storage, which occurs downstream of RAGE activation [136].

#### 3.4.1 Functional Implications of RAGE-ROS-calcium Axis

Highly reactive chemicals known as reactive oxygen species (ROS) and reactive nitrogen species (RNS) have a major impact on human disorders including cancer and cardiovascular disease [137]. ROS production and RAGE activation are linked, and this interaction plays a role in a number of cellular responses and pathological situations. It has been demonstrated that RAGE activation increases the activity of NADPH oxidase, a significant generator of ROS within cells [138]. Superoxide anions (O2•-) and other reactive oxygen species are produced when NADPH oxidase is activated [139]. Moreover, RAGE signaling has an impact on mitochondrial activity, which can raise ROS generation from the electron transport chain in the mitochondria [140]. This raises the total amounts of ROS in the cell. RAGE-mediated production of reactive oxygen species (ROS) is linked to NADPH oxidase (NOX) proteins such NOX1 and NOX2 [141]. The regulated production of reactive oxygen species (ROS) in reaction to RAGE activation is caused by these enzymes [141]. ROS thereby activates redox-sensitive transcription factors, such as Nuclear Factor-κB (NF-κB) and Activator Protein-1 (AP-1) [62]. These transcription factors play crucial roles in the regulation of inflammatory gene expression. Thus the pathophysiology of chronic illnesses, such as diabetes [142], neurological disorders [62], cardiovascular diseases [135], and inflammatory conditions, is linked to the interaction between ROS and RAGE activation [143].

#### 3.4.2 ROS measurement assays and their benefits

Understanding oxidative stress and its consequences in a range of biological processes and disorders requires an assessment of the reactive oxygen species (ROS) synthesized during the pathogenesis. ROS assessment will indirectly measure the effect of RAGE activation as well as the differential RAGE activity. Quantifying ROS levels can be performed in several ways, each having pros and cons. One such method is chemiluminescence assays, which uses chemiluminescent probe, such as luminol or lucigenin [144]. As ROS reacts with a chemiluminescent agent, the tests quantify the light released during this process. Chemiluminescence assays are useful tools for evaluating oxidative stress and cellular redox state in molecular biology because they offer distinct benefits over other methods for measuring reactive oxygen species (ROS) [145]. Chemiluminescence tests are highly sensitive and flexible making it possible to identify even low concentrations of reactive oxygen species (ROS), which are essential for researching alterations in oxidative stress in cells [146]. They enable comparisons between samples and experimental settings and offer precise measurement of ROS levels. Chemiluminescent probes provide specificity by allowing them to target certain ROS, such hydrogen peroxide or superoxide [147]. They have a high signal-to-noise ratio because of their minimal background signals, which makes them perfect for low-level noise measurements. Since chemiluminescent signals don’t require constant excitation, photobleaching is limited and signal intensity is maintained. They also simplify experimental settings by producing light without the need for outside light. The various kits available are listed in Table 2

Reactive oxygen species (ROS) are crucial for both normal physiological functions and disease states in the body. In cellular processes such as proliferation, differentiation, and apoptosis, ROS play crucial functions as signaling molecules. Through their involvement in redox signaling pathways, they alter the expression of genes and transcription factors. They are produced by immune cells and play a crucial role in homeostasis, antioxidant defense, and cellular redox balance. Primary byproducts of mitochondrial respiration, ROS are involved in signaling, energy generation, and cellular metabolism. Overproduction of reactive oxygen species (ROS) or inadequate antioxidant defenses can result in oxidative stress, which damages proteins, lipids, and DNA and can lead to disorders including cancer, diabetes, cardiovascular disease, neurodegenerative disorders, and aging. Prolonged inflammation increases the formation of ROS, which damages tissue and accelerates the course of inflammatory diseases. Oxidative stress levels in cells, tissues, and biological fluids may be measured by ROS analysis. Assessing ROS levels helps to assess the harmony between the generation of ROS and antioxidant defense systems.Among them, the two easy accessible, sensitive, and cost-effective methods for analyzing ROS production are 2’,7’-dichlorodihydrofluorescein diacetate (DCFH-DA) assay [157] and dihydroethidium (DHE) staining [158]. DCFH-DA labeling can measure ROS formation following chemical treatment, inflammatory signaling activation, or genetic mutation [159]. DCF is mostly measures hydroxyl radicals, peroxynitrite, and hydrogen peroxide, among other ROS [160]. This makes it possible to measure oxidative stress due to various causes, though cannot differentiate between them. It is adaptable for researching oxidative stress under different experimental settings as it may be used to measure overall ROS levels in cells and tissues [160]. Fluorescence microscopy may be used to visualize DCF, which gives spatial information regarding the distribution of ROS within cells and tissues. Reactive oxygen species (ROS) are produced by various cellular compartments, including mitochondria, endoplasmic reticulum (ER), and peroxisomes. Mitochondria are the primary source of ROS during aerobic respiration, producing byproducts of the electron transport chain. Fluorogenic probes MitoSOXTM Red and MitoTracker Red CM-H2XRos are used to detect ROS. Fluorescent dyes like dihydroethidium (DHE) can also be used to quantify mitochondrial ROS. ER-specific probes like ER-Tracker Red, Blue White DPX, and CellROX Green can find ROS inside the ER compartment. The unfolded protein response (UPR) pathway’s activity is measured using the FRET-based ER stress sensor. Peroxisomes, involved in hydrogen peroxide synthesis and metabolic activities, can be identified using dyes CellROX Orange and DCF-DA. Real-time monitoring of hydrogen peroxide level fluctuations can be achieved using the genetically encoded fluorescent probe HyPer.

Unlike the DCF approach, dihydroethidium (DHE) is used to assess in situ ROS production [161]. Using an epifluorescence-equipped microscope and a digital camera, the tissues stained with the dye are recorded. The fluorescence measured at 585 nm long-pass filter, and the number of ethidium bromide-labeled nuclei that directly measured the ROS intensity are counted using ImageJ software. Moreover, DHE assay is compatible with flow cytometry, mitochondrial oxidative stress, and live-cell imaging methods. It helps characterize subpopulations within a sample by identifying cells that produce more superoxide [162].

### 3.5 Importance of pro-inflammatory cytokine release after RAGE activation

Pro-inflammatory cytokines are released as a result of intracellular signaling pathways being activated by RAGE when coupled with ligands such as AGEs, S100 proteins [120], and HMGB1 [163]. These cytokines, which include TNF-α, IL-1β, and IL-6, play a crucial role in amplifying the inflammatory response, promoting the recruitment and activation of immune cells [163]. Pro-inflammatory cytokines released during RAGE-mediated inflammation contribute to the recruitment of immune cells, such as macrophages, neutrophils, and T cells, to the site of inflammation [164]. They also contribute to tissue damage and remodeling, potentially leading to chronic inflammation and tissue damage [163]. RAGE activation induces the activation of intracellular signaling pathways, including NF-κB and MAK pathways, which lead to the transcription and release of pro-inflammatory cytokines [163]. Dysregulation of RAGE-mediated persistent inflammation and its associated cytokine release has been linked to the pathogenesis of chronic diseases like diabetes, cardiovascular diseases, neurodegenerative disorders, and inflammatory autoimmune conditions [165]. Cytokines also contribute to the crosstalk between different cell types involved in the inflammatory response, creating a complex network that influences inflammation progression [165]. Modulation of immune responses by cytokines released during RAGE-mediated inflammation can have both protective and detrimental effects depending on the milieu [165]. List of proinflammatory cytokines activated during RAGE activations are listed in Table 3.

To comprehend the scope and dynamics of the inflammatory response, pro-inflammatory cytokine production during RAGE-mediated inflammation must be measured. Immune cell activation state and cytokine levels may be determined using real-time quantitative PCR for cytokine mRNA quantification and enzyme-linked immunosorbent assay (ELISA), Western Blotting, and immunohistochemistry for cytokine protein determination for cytokine protein production determination.

#### 3.5.1 Quantitative techniques to assess the proinflammatory cytokines.

Quantitative Q-PCR may detect mRNA expression of cytokines at their transcription levels from small amounts of samples. Even though it is cost effective, its drawbacks include need to isolate various cell types, and the inability to cross the threshold for detection in situations when only a small percentage of cells release the cytokines. It is a delicate test that necessitates cautious experimental design, implementation, and validation due to technical constraints such template quality, operator variability, the reverse transcription phase, and subjectivity in data processing and reporting [191]. An effective technique for precisely quantifying nucleic acids in a sample is digital polymerase chain reaction, or dPCR [192]. When working with limited or vital samples, when precision is crucial, it is very helpful. Digital PCR (dPCR) is a technique that partitions a sample into thousands of individual reactions to provide absolute quantification of target nucleic acids, with higher precision and sensitivity compared to traditional PCR [193]. It’s particularly useful when dealing with small sample quantities or low concentrations of targets, as it’s less susceptible to variations in amplification efficiency or sample quality [194]. Additionally, analyzing multiple samples can provide a more comprehensive understanding of target nucleic acid concentration variability, leading to more reliable conclusions in diagnostic assays [195]. Cytokine detection and quantification depend heavily on the digital PCR (dPCR) [196]. A single-molecule resolution offered by dPCR makes it possible to identify and measure individual cytokine molecules, which is very helpful for studying the heterogeneity of cytokine expression and finding uncommon cytokine-producing cells [197]. Furthermore, the multiplexing capabilities of dPCR make it possible to quantify many cytokines simultaneously in a single reaction, offering thorough insights into immune system activity and cytokine signaling pathways [198].

NanoString is another technological tool for understanding complex biological processes [199]. This powerful method enables simultaneous high-throughput study of copy number variations, miRNA levels, gene expression, and protein expression [199]. Its great sensitivity, accuracy, and adaptability make it perfect for a variety of uses, ranging from clinical diagnostics to fundamental research. Because nanostring can profile numerous targets at once, it’s a useful tool for studying complicated biological processes, finding biomarkers, and creating specific therapy strategies [200]. NanoString technology offers several benefits for cytokine quantification, including multiplexing capability, high sensitivity and specificity, unbiased quantification, flexible assay design, digital counting technology, and compatibility with various sample types [201].

Another technique that can detect released cytokines at the protein level is ELISA. It has drawbacks as well, such the challenge of getting enough tissue fluids and the possibility of underestimating real cytokine levels because of cytokine consumption by cells. Additionally, this technique makes it possible to identify small concentrations of cytokine-producing cells in a tissue that may not release enough cytokines to be picked up by other techniques. However, this approach might have limited sensitivity for protein secretion detection, and it is not as quantitative as the previous three methods. An alternative method for determining cytokine mRNA species is in situ hybridization on paraffin or freshly frozen tissue slices. This method will identify the types of cells that produce cytokines and where they are found. But like immunohistochemistry, this technique lacks quantitative capability, and the presence of RNA is not always indicative of the presence of protein[202]. One useful method for determining cytokine levels is flow cytometry, which offers many ways to measure cytokines in cell culture supernatants or at the single-cell level [203].

Using flow cytometry, intracellular cytokine staining is utilized to examine the expression of cytokines like interleukin-2 (IL-2), tumor necrosis factor-alpha (TNF-α), and interferon-gamma (IFN-γ) [204]. The analysis of cytokine expression among various immune cell subsets, such as CD4+ T cells, CD8+ T cells, and natural killer cells, is therefore done using flow cytometry [205]. This process offers insights into the distinct cytokine-producing cell populations implicated in the antiviral immune response. Another novel approach to studying proteins in biological fluids such as blood, urine, saliva, and cerebrospinal fluid is the use of liquid tissue [206]. In liquid tissue studies, samples are taken, and their proteins are identified and quantified by analysis. Non-invasive sampling, thorough protein profiling, dynamic and varied samples, and the possibility of clinical application are some advantages of liquid tissue research. All things considered, liquid tissue research might lead to breakthrough discoveries in patient diagnosis and treatment.

### 3.6 RAGE and apoptosis

Chronic inflammation has been shown to contribute to cell death, and RAGE activation is frequently associated with inflammatory responses. RAGE-mediated signaling has the potential to trigger pro-inflammatory pathways and, under specific conditions, result in inflammatory-induced cell death [10]. Apoptosis is an essential step for proper growth and tissue homeostasis [207], and RAGE plays a significant role in it [208]. By inducing either apoptosis or survival, its signals can affect cell fate decisions [208]. RAGE-mediated apoptosis has been connected to neurological disorders like Alzheimer’s and may be engaged in physiological functions or cell removal [209]. The extrinsic apoptotic pathway, which involves death receptors including Fas (CD95) and tumor necrosis factor receptor 1 (TNFR1) [210], can also be triggered by RAGE-AGE interaction [211]. This interaction can affect mitochondrial function and cause oxidative stress [211]. Pro-apoptotic substances, such cytochrome c, can be released from the mitochondria when there is a disruption in the integrity and function of the mitochondria [212]. This leads to the activation of downstream effector caspases via the activation of caspase-8 [212].Cytochrome c release triggers the intrinsic apoptotic process by activating caspases, namely caspase-9 [213]. Caspase-9 triggers caspase-3, -6, and -7, which are downstream effector caspases that cleave different cellular substrates and eventually cause apoptosis in cells [214]. The article discusses a number of approaches for evaluating cell death.

#### 3.6.1 Quantitative assays for assessing cellular viability and apoptosis rates in response to RAGE activation

Several quantitative assays that are commonly used to assess cellular viability and apoptosis rates in response to RAGE activation are MTT assay, CCK-8 assay, PI and Annexin V staining for flow cytometry, TUNEL assay, caspase activity assays, DNA fragmentation assay, real-time cell analysis, and LDH release assay. Among the cell viability assays, the most widely used cell viability assay is MTT assay [215]. It is the colorimetric method for assessing cellular viability and proliferation, offering quantitative measurement, high sensitivity, ease of use, cost-effectiveness, versatility, and compatibility with high-throughput screening. Even though it is compatible with both adherent and suspension cells, the potential limitations include interference from compounds, the need for a viable cell population, and inability to distinguish between proliferating and non-proliferating cells. The MTT assay measures cell viability and metabolic activity but can be affected by certain compounds or experimental conditions, leading to inaccurate results. To obtain reliable results, careful experimental design and validation are necessary [216]. The assay relies on viable cells, so it may not be accurate if a significant proportion of non-viable or apoptotic cells are present. The assay does not distinguish between proliferating and non-proliferating cells, so additional assays may be required to provide a more complete assessment of cellular responses [216]. Hence, a variety of factors influence the results of the MTT test. To prevent obtaining false conclusions about the viability of cells, the toxicity of treatments, and the metabolism of cells, the assay must be optimized, and the data must be carefully interpreted.

Compared to MTT assay, the CCK-8 assay provides several advantages including reduced incubation time, direct quantification, high sensitivity and linearity, broad dynamic range, a single-step process, no toxicity to cells, compatibility with multimode readers, and stable formazan product [217]. Additionally, the CCK-8 assay generates a measurable signal within 1–4 hours, enabling quicker data acquisition [218]. Furthermore, it is compatible with live cells and does not require solubilization steps, simplifying the assay procedure. The formazan product generated by the CCK-8 assay is water-soluble, allowing for direct quantification without solubilization solutions, and reducing the likelihood of experimental errors [218]. Overall, these studies provides valuable insights into RAGE signaling and cellular survival and utilizes a reliable and efficient method for assessing cell viability.

Flow cytometry is a widely used method to assess cell death, particularly in the context of RAGE-mediated cell death [219]. The combination of Propidium Iodide (PI) staining and Annexin V staining categorizes cells into live, early apoptotic, late apoptotic, and necrotic populations. An increase in Annexin V-positive cells indicates apoptotic pathway activation [220]. The specificity of ligands used to activate RAGE is crucial, as RAGE signaling can influence various cell death pathways. Additional cell death assays can confirm findings for a more detailed understanding of cell death mechanisms. TUNEL assay provides visual and quantitative information, caspase activity assays directly measure key enzymatic events, and DNA fragmentation assays offer insights into the later stages of apoptosis [221]. Detects DNA fragmentation, a characteristic feature of apoptosis [222]. The list of cell death assay and its advantages are listed in Table 4.

### 3.7 RAGE and mitochondrial membrane potential

Mitochondrial dysfunction is a well-known contributor to cell death, which can occur through either apoptosis or necrosis [227]. Recent discoveries have shown that RAGE activation is associated with oxidative stress and inflammation [228], plays a significant role in mitochondrial dynamics, respiration, and oxidative stress [228]. The opening of the MPTP (mitochondrial permeability transition pore) is a critical event that leads to mitochondrial dysfunction and is linked to various forms of cell death, including apoptosis and necrosis [229]. The MPTP opening can be triggered by various conditions, such as cellular stress, injury, and increased reactive oxygen species (ROS) production [230], and can result in the collapse of the mitochondrial membrane potential (ΔΨm) and the release of pro-apoptotic factors [230]. Calcium influx and RAGE activation can also impact mitochondrial function by sensitizing mitochondria to MPTP opening [231]. The increased cytosolic and mitochondrial calcium levels, driven by RAGE signaling, also contribute to MPTP opening [231]. ROS can modulate proteins involved in MPTP regulation, and phosphate can interact with mitochondrial proteins and contribute to the regulation of MPTP [232].

Various techniques, such as flow cytometry, spectroscopy, and fluorescence microscopy, can be employed to assess mitochondrial function by monitoring changes in ΔΨm [233], calcium retention capacity [234], or the release of mitochondrial proteins [235]. The MPTP assay provides a valuable tool to investigate the role of RAGE in mitochondrial permeability transition and its contribution to cellular responses [236]. It is essential to complement the results from the MPTP assay with other assessments of mitochondrial function, such as ΔΨm measurements or evaluations of mitochondrial protein release. Additional assays, such as apoptosis markers or caspase activity assays, can confirm the occurrence of cell death.

Cell lines or primary cells expressing RAGE or exposed to RAGE ligands can be used to investigate the impact on mitochondrial function. Various agents, such as calcium ionophores or oxidative stress inducers, can be used to modulate MPTP opening [237]. MPTP inhibitors can be employed to assess the role of MPTP in RAGE-mediated effects [238]. The use of specific RAGE inhibitors or activators, along with assessments of MPTP opening, can provide insights into the causal relationship between RAGE activation and mitochondrial permeability transition.

In summary, RAGE activation impacts mitochondrial membrane potential and function, leading to cell death. The opening of MPTP, which is associated with RAGE activation, can result in mitochondrial swelling, rupture, and the release of pro-apoptotic factors. The MPTP assay provides a valuable tool to investigate the role of RAGE in mitochondrial permeability transition and its contribution to cellular responses, shedding light on the mechanisms underlying RAGE-mediated cell death. The use of specific RAGE inhibitors or activators, along with assessments of MPTP opening, can provide insights into the causal relationship between RAGE activation and mitochondrial permeability transition, which can help identify new targets for therapeutic interventions.

## 4. Future Directions

RAGE is a transmembrane receptor protein that has been implicated in various pathological and physiological processes. It plays a crucial role in the development and progression of several diseases such as diabetes, Alzheimer’s, and cancer, by inducing chronic inflammation and contributing to tissue damage. Therefore, the inhibition or attenuation of RAGE signaling has become a promising therapeutic option. siRNA and shRNA are two techniques that can be used to inhibit RAGE activity. They are designed to specifically target and silence the RAGE gene, resulting in the degradation of RAGE mRNA and the prevention of RAGE protein synthesis. This approach has been shown to effectively attenuate RAGE signaling in different experimental models, making it a promising strategy for treating RAGE-related diseases.

A potential approach for creating innovative treatments to reduce the harmful consequences of RAGE activation in various disorders is the design of small molecule inhibitors that target the binding interface between RAGE and its ligands, such as AGEs, HMGB1, and S100 proteins. Designing small compounds that obstruct RAGE-ligand interactions can be achieved by high-throughput screening and structure-based drug design techniques. The activation of RAGE and subsequent signaling can be stopped by small compounds that either block the ligand-binding site on RAGE or imitate the structure of RAGE ligands. Soluble RAGE, or sRAGE, is one such substance that is now in use. Its specific function is to suppress RAGE-mediated signaling pathways.

Another possible strategy is to create small molecule inhibitors that specifically target significant signaling molecules such JAK/STAT pathways, NF-κB, MAPKs, and PI3K/Akt pathways that occur downstream of RAGE activation. It is possible to find small compounds that specifically block RAGE-mediated signaling pathways while leaving physiological signaling pathways unaffected by screening compound libraries or applying computational methods. By interrupting the pro-survival and inflammatory signaling cascades brought on by RAGE activation, these inhibitors can decrease the pathogenic consequences of RAGE signaling. Preclinical studies have shown that these drugs have promising therapeutic potential, and some of them are currently being evaluated in clinical trials. By targeting RAGE-ligand interaction and downstream signaling using novel small drugs, it is possible to develop better therapeutics for patients suffering from RAGE-related diseases.

Therefore, the creation of small-molecule drugs that target downstream signaling pathways and RAGE-ligand interactions holds enormous potential for translational research and the development of novel therapeutics for the treatment of RAGE-associated diseases, such as cancer, neurodegenerative diseases, chronic inflammatory conditions, and complications from diabetes.

## 5. Conclusion

RAGE-ligand binding activates various intracellular signaling pathways, contributing to cellular responses in inflammation, oxidative stress, and immune reactions. The MAPK/ERK, PI3K/Akt, and JAK/STAT pathways are the major intracellular signaling cascades triggered by RAGE activation. RAGE activation is associated with various inflammatory-related clinical conditions, including diabetes, cancer, vascular disease, and neurodegeneration. Quantitative assessment techniques, such as Mass Spectrometry (MS), Phosphoproteomics Antibody-Based Techniques such as western blotting, immunoprecipitaion (IP) and immunofluoresence microscopic examination and high-throughput screening of protein interactions and modifications by protein microarrays and site-directed mutagenesis along with classic PCR, real-time PCR, are employed to assess RAGE activation under different inflammatory conditions.

In order to provide precise and efficient therapies for diseases associated with RAGE dysregulation, further study is needed to completely understand the function of RAGE in disease evaluation and treatment. Therefore, translational research and the development of novel therapeutics for the treatment of RAGE-associated diseases, including cancer, neurodegenerative disorders, and chronic inflammatory conditions, are greatly encouraged by the discovery of small molecule drugs that target RAGE-ligand interactions and downstream signaling pathways.

## Figures and Tables

**Table 1:** Function of RAGE in different clinical conditions

Molecular mechanism	Pathology	Disease/clinical condition
Oxidative stress	Neurological Inflammation	• Alzheimer’s Disease-Increased inflammation and oxidative stress [20] • Parkinson’s Disease – Increased neuronal damage[21] • Stroke - Inflammatory and neuronal injury[22] • Multiple Sclerosis (MS) - Demyelination and axonal damage[23] • Amyotrophic Lateral Sclerosis - Neuroinflammation and motor neuron degeneration[24]. • Diabetic Neuropathy - Nerve damage and sensory deficits[25]
Angiogenesis	Pulmonary Disorders	• Chronic Obstructive Pulmonary Disease - recruitment of inflammatory cells and the destruction of lung tissue[3] • Asthma - Increased airway inflammation, mucus production, and airway hyper responsiveness[26] • Pneumonia - Increased recruitment of immune cells and the release of pro-inflammatory cytokines[27] • Acute lung injury – Increased inflammatory response and endothelial dysfunction[28]. • Pulmonary hypertension – Increased vascular remodeling and inflammation in the pulmonary arteries[29]. • Lung cancer - Activate signaling pathways that enhance cancer cell survival, proliferation, and invasiveness[30].
Proliferation	Cardiovascular Diseases	• Atherosclerosis – Increased inflammatory responses and the recruitment of immune cells to the arterial wall. Plaque formation and destabilize atherosclerotic lesions[31] • Cardiac fibrosis – Contribute to increase deposition of extracellular matrix proteins in the heart tissue • Hypertension - Contribute to vascular dysfunction and remodeling, leading to increased blood pressure[32]. • Diabetic Cardiovascular Complications[33] • Vascular Calcification –Increased calcium deposits accumulation in the arterial walls[33, 34]. Myocardial Infarction - Contribute to the progression of heart failure by impairing cardiac function[31].
RAGE expression	Digestive diseases	• Inflammatory Bowel Disease (IBD) - Contribute to the inflammatory response and tissue damage in the gut[35]. • Gastric Cancer - Promote tumor growth and metastasis in gastric cancer[36]. • Liver cancer – Increased inflammation and fibrosis in the liver. • Pancreatitis - Involved in acute and chronic pancreatitis[37] • Colorectal Cancer- Associated with increased tumor progression and invasion in the colon[38]
Inflammation	Diabetic and metabolic diseases	• Type 2 Diabetes - Contributes to insulin resistance and the development of diabetic complications[39, 40] • Insulin Resistance - Impair insulin signaling pathways, contributing to reduced glucose uptake by cells[41]. • Obesity – Increased expression in adipose tissue lead to increased chronic inflammation and insulin resistance[42]. • Non-Alcoholic Fatty Liver Disease (NAFLD) - Contributes to inflammation, fibrosis, and the progression of NAFLD[43] • Dyslipidemia and hypertension - Interact with oxidized lipids and induces oxidative modifications[31].
Migration	Urological Disorders	• Diabetic Nephropathy - Recruitment of immune cells, endothelial dysfunction, and increased permeability of the glomerular filtration barrier and contributing to proteinuria and kidney damage[44, 45] • Acute kidney injury - Contribute to inflammation and cell death on acute kidney injury[46] • Prostate cancer - Increased cell proliferation and survival in cancer cells[47] • Bladder cancer - Play a role in tumor invasion and metastasis, influencing processes such as epithelial- mesenchymal transition (EMT) and interactions with the extracellular matrix[48]. • Hypertensive nephropathy – Increased deposition of extracellular matrix proteins and fibrosis in the renal tissue[49]

**Table 2:** List of commercially available assay kits for chemiluminescence that are intended to detect reactive oxygen species (ROS) in tissues and cells

Reagent	Manufacturer	Application	Principle	References
CellROX^®^ Deep Red Reagent	Thermo Fisher Scientific	Detection of a broad range of ROS, including superoxide and hydroxyl radicals.	CellROX^®^ reagents become highly fluorescent upon oxidation by ROS and can be detected using fluorescence or chemiluminescence.	[148]
DCFDA Cellular ROS Detection Assay Kit	Abcam	Measurement of general cellular ROS levels.	DCFDA is oxidized by ROS to form the fluorescent compound DCF, which can be detected using fluorescence or chemiluminescence.	[149]
Superoxide Anion Detection Kit	Enzo Life Sciences	Specifically detects superoxide anions.	Utilizes chemiluminescent probes to react with superoxide, producing a chemiluminescent signal.	[150]
Hydrogen Peroxide Assay Kit	Abcam	Quantification of hydrogen peroxide levels	Chemiluminescent reaction with luminol in the presence of hydrogen peroxide, producing light emission	[151]
OxiSelect^™^ In Vitro ROS/RNS Assay Kit	Cell Biolabs, Inc	Detection of ROS and reactive nitrogen species (RNS)	Employs a chemiluminescent substrate to detect a broad spectrum of ROS and RNS	[152].
Luminol Chemiluminescence Assay Kit	BioVision	General detection of ROS	Luminol reacts with ROS to produce a chemiluminescent signal that can be measured	[153]
ROS-ID^®^ Total ROS/Superoxide Detection Kit	Enzo Life Sciences	Simultaneous detection of total ROS and superoxide	Chemiluminescent probes are used to detect both total ROS and superoxide levels	[154]
Amplex Red Hydrogen Peroxide/Peroxidase Assay Kit	Thermo Fisher Scientific	Quantification of hydrogen peroxide levels	Amplex Red reacts with hydrogen peroxide in the presence of peroxidase to produce a fluorescent or chemiluminescent signal	[155]
ROS-Glo^™^ H2O2 Assay	Promega	Detection of hydrogen peroxide.	Utilizes a luminogenic substrate to quantify hydrogen peroxide levels through a chemiluminescent reaction.	[156]

**Table 3:** Proinflammatory cytokines activated during RAGE activations

Cytokines	Functions in RAGE pathway	References
Interleukin-1β (IL-1β)	• Recruitment of inflammatory cells such as neutrophils and monocytes to the site of inflammation. • Inducing the expression of adhesion molecules, chemokines, and other cytokines • Inducing the production of matrix metalloproteinase (MMPs) and other enzymes involved in tissue remodeling. • Promoting the differentiation and activation of T cells	[166] [167] [168] [169]
Tumor Necrosis Factor-alpha (TNF-α)	• Stimulating various immune (such as macrophages and neutrophils) and non-immune cells. • Contributing to the initiation and propagation of inflammatory signaling cascades. • Inducing vasodilation and increasing vascular permeability. • Contributing to cell death and tissue injury. • Influencing the activation and function of T cells.	[170] [171] [172] [173] [2]
Interleukin-6 (IL-6)	• Inducer of the acute phase response, leading to the synthesis of acute-phase proteins • Stimulating hepatocytes to produce acute-phase proteins such as C-reactive protein (CRP) • Inducing the differentiation and function of T cells and modulate adaptive immune responses • Modulating endothelial function and promoting angiogenesis • Contributing to tissue alterations and repair mechanisms.	[174] [175] [176] [177] [178]
IL-8 (CXCL8)	• Promoting the recruitment of neutrophils to the site of inflammation • Contributing to angiogenesis • Inducing the expression of adhesion molecules on endothelial cells, facilitating the adhesion of immune cells to the vascular endothelium	[179] [180] [181]
Monocyte Chemoattractant Protein-1 (MCP-1)	• Recruitment of monocytes • Induces accumulation of cholesterol and immune cells in arterial walls and contribute to the development and progression of atherosclerotic plaques.	[182] [183]
Interleukin-17 (IL-17)	• Produced by T helper 17 (Th17) cells and enhancing the inflammatory response • Recruiting immune cells, especially neutrophils, to the site of inflammation • Enhancing antimicrobial responses, particularly against extracellular pathogens	[184] [185] [186]
Interferon-gamma (IFN-γ)	• Contribute to the polarization of immune responses toward a Th1 • Contribute to the activation of macrophages, leading to enhanced phagocytosis and production of inflammatory mediators	[187] [188]
Transforming Growth Factor-beta (TGF-β)	• Acting as both a pro-inflammatory and anti-inflammatory cytokine in a context-dependent manner • A key regulator of tissue repair and remodeling	[189] [190]

**Table 4:** Advantages and disadvantages of different RAGE specific cell death assays

Assay	Advantages	Disadvantages	References
PI (Propidium Iodide) Staining	• Used to identify necrotic cells based on their increased membrane permeability • A simple and quick assay for distinguishing live and dead cells	• PI staining does not differentiate between apoptotic and necrotic cell death. • It is not specific to particular cell death pathways	[223]
Annexin V Staining	• Annexin V is used to detect apoptotic cells by binding to phosphatidylserine exposed on the outer leaflet of the plasma membrane during early apoptosis • Allows discrimination between early apoptotic, late apoptotic, and necrotic cells	• It may not accurately distinguish between apoptosis and other forms of cell death. • The method may yield false positives or negatives in certain conditions.	[224]
TUNEL (Terminal deoxynucleotidyl transferase dUTP nick end labeling) Assay	• TUNEL detects DNA fragmentation, a characteristic feature of apoptosis. • It provides information on the late stages of apoptosis	• It may not be specific for apoptosis as DNA fragmentation can occur in other forms of cell death. • False positives may occur due to DNA damage unrelated to apoptosis.	[224]
Caspase Activity Assays	• Caspases are key enzymes in the apoptotic pathway, so their activity assays can specifically indicate apoptosis. • Various caspase substrates allow detection of different caspase activities.	• Some cells may undergo caspase-independent cell death pathways. • Caspase activation does not exclusively indicate apoptosis.	[225]
DNA Fragmentation Assay	• Detects DNA fragmentation associated with apoptosis. • Can be used to assess the later stages of apoptosis	• Similar to the TUNEL assay, it may not be entirely specific for apoptosis. • May miss early apoptotic events	[226]

## Abbreviations:

RAGE: Receptor for Advanced Glycation Endproducts
AGE: Advanced Glycation Endproducts
ENRAGE: Elevated RAGE
SRAGE: Serum RAGE
MAPK: Mitogen-activated protein kinases
ERK: Extracellular signal-regulated kinase
PI3K: Phosphoinositide 3-kinases
Akt: protein kinase B
JAK: Janus kinase
STAT: Signal Transducer and Activator of Transcription3
NF-κB: Nuclear factor kappa-light-chain-enhancer of activated B cells
HMGB1: High mobility group box 1
LPA: Lipoprotein
Aβ: Amyloid beta peptide
API: Activator protein 1
NADPH: Nicotinamide adenine dinucleotide phosphate
PTM: Post-translational modifications
TRP: Transient receptor potential
CaMK: Calmodulin-dependent protein kinase
IP3: Inositol trisphosphate
PLC: Phospholipase C
CREB: cAMP Response Element-Binding
ROS: Reactive Oxygen Species
DCFH-DA: 2’,7’-dichlorodihydrofluorescein diacetate
DHE: Dihydroethidium
TNF-α: Tumor necrosis factor alpha
IL6: Interleukin 6
IL4: Interleukin 6
MCP1: Monocyte Chemoattractant Protein-1
IFN-γ: Interferon-gamma
TGF-β: Transforming Growth Factor-beta
ELISA: enzyme-linked immunosorbent assay
MPTP: mitochondrial permeability transition pore
